# EUS–guided fine-needle aspiration for the diagnosis of hepatic metastatic neuroendocrine tumor (with videos)

**DOI:** 10.1097/eus.0000000000000146

**Published:** 2025-11-03

**Authors:** Sashuang Wang, Yating Wang, Dongqiang Zhao

**Affiliations:** Department of Gastroenterology, The Second Hospital of Hebei Medical University, Hebei Key Laboratory of Gastroenterology, Hebei Institute of Gastroenterology, Hebei Clinical Research Center for Digestive Diseases, No. 215 He Ping West Road, Xinhua District, Shijiazhuang 050000, Hebei Province, China.

A 75-year-old male presented with postprandial bloating, nausea, vomiting, and reduced bowel movements. Contrast-enhanced abdominal computed tomography (CT) revealed diffuse heterogeneous small intestinal wall thickening with luminal narrowing in the left abdomen, along with multiple hepatic hypodense lesions, suggestive of small intestinal neoplasm with liver metastases [Figure 1]. Tumor marker tests showed CA-125 40.99 U/mL and CA-199 52.43 U/mL. To establish a definitive diagnosis, endoscopic ultrasound-guided fine-needle aspiration (EUS-FNA) was performed.^[1]^ EUS demonstrated a 2.0 × 2.0-cm hypoechoic lesion in the left hepatic lobe, characterized by hyperechoic margins and homogeneous internal echoes [Figure 2A]. Elastography demonstrated blue-green coloration [Figure 2B]. Contrast-enhanced EUS using 3 mL intravenous sulfur hexafluoride microbubbles revealed progressive enhancement starting 10 seconds post-injection, with an additional 1.0 × 0.8-cm enhancing lesion detected distally [Figure 2C, Video 1]. EUS-FNA was performed via transgastric approach using a 19G needle (COOK EchoTip Ultra), with two passes (10 actuations per pass) yielding tissue and cellular material [Figure 3, Video 2]. No postprocedural complications occurred. Histopathology (HE staining) confirmed malignant cells. Immunohistochemistry showed positivity for CK8/18, CKpan, and Syn, with a Ki-67 index of 70% [Figure 4]. A diagnosis of grade 3 neuroendocrine tumor (NET G3) was established. Given the high proliferative activity of G3 NET and evidence of metastatic disease, the patient was referred for systemic oncologic treatment.

**Figure 1:**
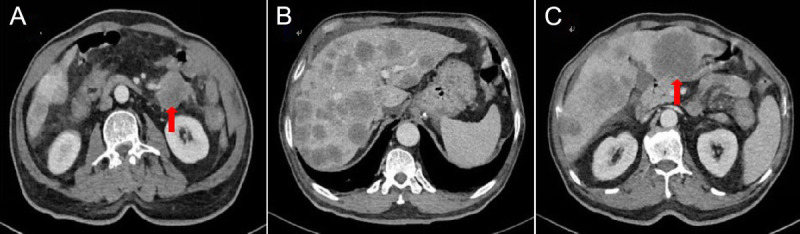
Contrast-enhanced abdominal CT revealed diffuse heterogeneous wall thickening of the left-sided jejunal bowel loops with luminal narrowing (A), suspicious for malignant infiltration, along with multiple hypodense space-occupying lesions in the liver consistent with metastatic involvement (B). The lesion in the left lobe of the liver adjacent to the stomach is the target of this EUS-FNA procedure (C).

**Figure 2:**
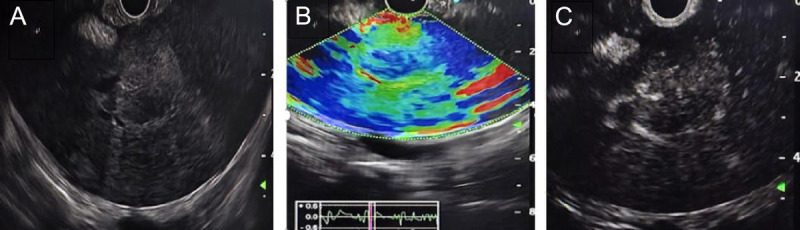
EUS (A) revealed a round, hypoechoic lesion (2.0 × 2.0 cm) in the left hepatic lobe with homogeneous internal echogenicity, bordered by a hyperechoic margin. Elastography (B) demonstrated a mixed blue-green coloration pattern. CEH-EUS (C) showed significant contrast enhancement.

**Figure 3:**
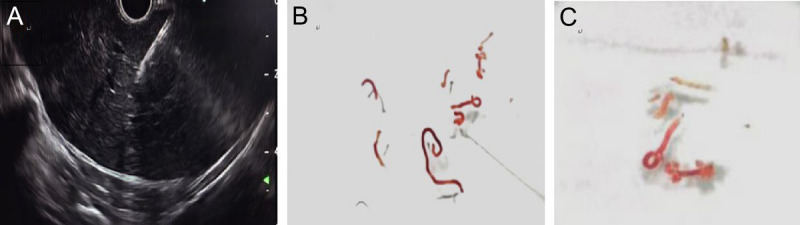
Transgastric EUS-FNA was performed using a 19-gauge FNA needle (A). Adequate tissue samples were obtained (B, C).

**Figure 4:**
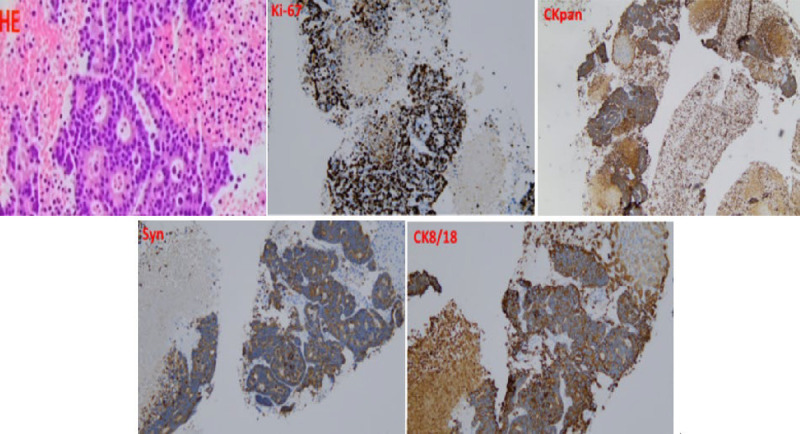
HE staining and immunohistochemical markers (Ki-67, synaptophysin, CKpan, CK8/18) were demonstrated.

Small intestinal neuroendocrine tumors (SI-NETs), classified as malignant neoplasms arising from enterochromaffin cells, predominantly occur in the ileum and less frequently in the jejunum. Recent epidemiological data indicate a global rise in the incidence of these tumors. Due to the small bowel’s deep anatomical location and nonspecific early symptoms, many patients are diagnosed with distant metastases at presentation, with the liver being the most common metastatic site.^[2]^ Although EUS-FNA is a well-established technique for sampling hepatic lesions,^[3]^ its application in diagnosing liver metastases from small intestinal neuroendocrine tumors (NETs) has not been previously described. This case report documents the first use of EUS-FNA to confirm small intestinal NET hepatic metastasis, marking a significant contribution to the precise diagnosis and treatment of small intestinal neuroendocrine tumor liver metastases.

## Supplementary Videos

**Video 1:** Multimodal EUS (elastography and contrast-enhanced imaging) delineates the location, structural features, stiffness profile, and vascular dynamics of the hepatic lesions in the left lobe;

**Video 2:** EUS-FNA of the target lesion was performed using a 19-gauge FNA needle. Videos are only available at the official website of the journal (www.eusjournal.com).

## Acknowledgments

None.

## Source of Funding

None.

## Ethical Approval

The case was approved by the institutional review board.

## Informed Consent

Written informed consent was obtained from the patient for the publication of this case report.

## Conflict of Interest

The authors state that there are no conflicts of interest regarding this article.

## Author Contributions

S. Wang conducted the literature review and was involved in drafting the manuscript and creating the video. Y. Wang was responsible for image acquisition. D. Zhao oversaw the conceptualization and supervised the project. All authors contributed to manuscript revision, read, and approved the submitted version.

## Data Availability Statement

The authors confirm that the data supporting the findings of this article are available within the manuscript.
